# Leveraging genomics to understand the broader role of fungal small secreted proteins in niche colonization and nutrition

**DOI:** 10.1038/s43705-022-00139-y

**Published:** 2022-06-28

**Authors:** Jonathan M. Plett, Krista L. Plett

**Affiliations:** 1 Hawkesbury Institute for the Environment, Western Sydney University, Locked Bag 1797, Penrith, NSW 2751 Australia; 2 Elizabeth Macarthur Agricultural Institute, NSW Department of Primary Industries, Menangle, NSW 2568 Australia

**Keywords:** Fungal biology, Symbiosis

## Abstract

The last few years have seen significant advances in the breadth of fungi for which we have genomic resources and our understanding of the biological mechanisms evolved to enable fungi to interact with their environment and other organisms. One field of research that has seen a paradigm shift in our understanding concerns the role of fungal small secreted proteins (SSPs) classified as effectors. Classically thought to be a class of proteins utilized by pathogenic microbes to manipulate host physiology in support of colonization, comparative genomic studies have demonstrated that mutualistic fungi and fungi not associated with a living host (i.e., saprotrophic fungi) also encode inducible effector and candidate effector gene sequences. In this review, we discuss the latest advances in understanding how fungi utilize these secreted proteins to colonize a particular niche and affect nutrition and nutrient cycles. Recent studies show that candidate effector SSPs in fungi may have just as significant a role in modulating hyphosphere microbiomes and in orchestrating fungal growth as they do in supporting colonization of a living host. We conclude with suggestions on how comparative genomics may direct future studies seeking to characterize and differentiate effector from other more generalized functions of these enigmatic secreted proteins across all fungal lifestyles.

## Introduction

**Fig. 1:**
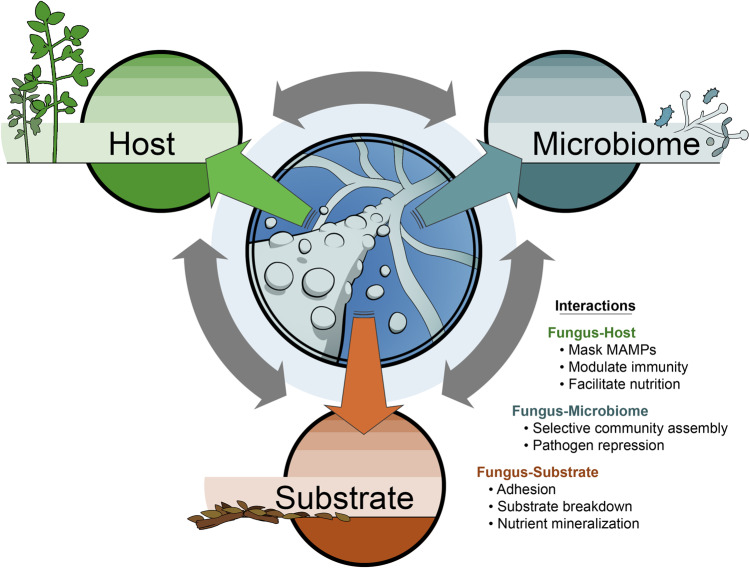
The fungal secretome influences numerous inter-dependent interactions with a plant host, the surrounding microbiome and the substrate. Some specific examples of interactions facilitated by secreted proteins are listed. MAMP stands for ‘microbe-associated molecular patterns’.

Plant-associated fungi of all lifestyles have adapted the means to colonize a particular niche, defined here as plant tissue (living or dead) and surrounding substrate (i.e., soil), including mechanisms of host communication, microbiome manipulation, and nutrient acquisition. These interactions are supported by a myriad of secreted proteins, together known as the fungal secretome (Fig. 1). An ever-increasing number of sequenced fungal genomes has greatly advanced understanding of both the complement and evolution of the fungal secretome across a breadth of lineages [1–5]. Secreted proteins, characterized by the presence of a secretion domain with no accompanying transmembrane domain, are estimated to encompass as much as 4–15% of proteins encoded in fungal genomes [6] and encompass a variety of enzymatic proteins and small secreted proteins.

Enzymatic proteins, including carbohydrate-active enzymes (CAZymes), proteases and lipases, are necessary for the breakdown of organic material to obtain nutrients or in modifying plant tissues during host interactions. The specific enzyme classes encoded in each fungal genome are partially influenced by the lifestyle of the fungus. Lignin and cellulose degradation enzymes are most abundant in the genomes of saprotrophic fungi whose environmental niche are dead and decaying organisms, while hemibiotrophic or necrotrophic pathogens have host-activated secretomes weighted towards the degradation of plant tissues [1, 4]. Typically, the genomes of biotrophic pathogens or mutualistic fungi, which require a living host niche throughout their lifecycle, have the lowest number of plant cell wall active enzymes. This is especially a hallmark in the evolution of ectomycorrhizal fungi where a strong reduction in the complement of CAZymes has been found in all genomes studied to date [5, 7, 8].

In addition to secreted enzymes, as much as 40–60% of the secretome consists of small secreted proteins (SSPs; often defined as <300 amino acids in size, although this is not an absolute cut-off for secreted effector proteins; 3,4). A sub-group of SSPs, typically characterized by high cysteine content and induced in the presence of a host organism, are classified as ‘effectors’: proteins used to modulate host defenses or physiology to promote colonization. Due to their role in manipulating the host to facilitate colonization, effector proteins were initially assumed to be characteristic of pathogenic fungi, however, effector proteins were also found in mutualistic mycorrhizal fungi and shown to have functions similar to those in pathogenic organisms [9, 10]. Candidate effector proteins have also been found in the genomes of fungi from all lifestyles, including saprotrophic fungi [11, 12]. Likewise, it was originally assumed that effectors were fast-evolving genes with minimal homology across species, however, the increased availability of sequenced fungal genomes has shown conservation of some of these sequences across a broader range of fungi than expected [5, 11, 13, 14]. While candidate effectors are more abundant in biotrophic fungi [4], the genomic content and evolution of these effectors are more likely to be influenced by lineage than lifestyle [2]. This raises the question of the broader role of these effector SSPs, particularly where they are conserved across very different fungal lifestyles [5].

In this review we will consider the roles of fungal effector proteins across a range of lifestyles, focusing primarily on fungi with a biotrophic component during their life cycle. Given mounting evidence for a role of effectors beyond plant host interactions, stemming both from evolutionary genomic evidence and new effector characterization studies, we will extend beyond the classic role of effectors in host compatibility into the emerging areas of microbiome structuring and nutrient acquisition.

## Effectors and colonization of the host niche

The role of secreted proteins as effectors during niche colonization of host plants by fungi has been the focus of a number of reviews (e.g., [11, 15, 16]), with our best understanding of how effector proteins function coming from studies involving pathogenic fungi. Broadly speaking, biotrophic SSPs involved in host colonization play a role in four main areas: hiding the fungus from host detection, modifying host physical characteristics, tuning host signaling pathways, and altering host enzymatic processes. In this section we will cover some of the highlights of recent research across the range of fungal lifestyles.

Over the course of evolutionary time, plants have developed a range of different means by which foreign organisms are detected including the perception of microbe-associated molecular patterns (MAMPs). One key class of MAMPs are chitin oligosaccharides, components of fungal cell walls that can be either bound or free. Therefore, one role of effector proteins expressed in the early stages of symbiosis is to ‘hide’ the presence of MAMPs from the host. This can be done by binding directly to chitin and chelating it [17], modifying the form of chitin [18], or degrading the free forms of the oligosaccharide [19]. A recent discovery of a novel pathway by which biotrophic pathogens use effector proteins to attenuate MAMP detection was brought about by the study of the effector SsPele1 of *Sporisorium scitamineum* [20]. This protein sequence mimics a plant protein enabling it to competitively bind to a plant MAMP receptor kinase thereby disrupting plant immune responses. Therefore, co-evolution of similar sequences between plant and fungi has enabled certain microbes to mask their presence from the host. Mutualistic fungi have also developed similar effectors to pathogenic fungi that are induced during symbiosis, disruption of which inhibits host colonization [21]. However, fungi produce dozens of other more general and lineage-specific MAMP-like elicitors, therefore more work is needed to identify how fungal effectors may be able to directly alter/mask the activity of these other elicitors. It is unlikely that every single elicitor class has a matching effector to hide its presence, which is why fungi also encode a fleet of effectors that can physically alter host tissues or manipulate host processes and defense signaling.

Following initial signaling exchange, fungi must adhere to, or invade, plant host tissues. While a range of adhesins or oligosaccharides can aid in the former step, new effectors are also being discovered that can modify the chemical properties of plant cell walls. For instance, a comparative genomics approach across different lifestyles of fungi within the Pezizomycotina identified a conserved sequence motif (C-CXXXC-C-C-C-C-C) within surface-active effectors [22]. These proteins, when tested, changed the hydrophobicity of a surface to promote hyphal attachment regardless of fungal lifestyle. Within mutualistic fungi, a range of secreted effector-like hydrophobins may also support attachment to host surfaces during colonization [23]. Effector SSPs can also physically modify the structure of plant cells, with a study in the mutualistic fungus *Laccaria bicolor* identifying a secreted glucosyl hydrolase (GH5) that had high activity against plant cell wall components, but not fungal cell wall components [24]. Transgenic knock-down of this protein resulted in an impaired ability of the fungus to grow within host tissues. While these two classes of surface-active proteins may not be considered classical effectors, they both modulate an aspect of host biology necessary to support microbial colonization. Therefore, as research begins into the very early stages of plant-fungal symbioses, surface-active effector proteins should become a focus as they are likely to have been one of the first innovations of fungi that evolved to intimately interact with a living substrate.

The plant immune system regulates pathways affecting hormone signaling, reinforcement of cell walls, or production of toxic secondary compounds. Identification of effectors that alter these pathways have remained the focus of a large portion of work performed to date in pathogenic and mutualistic fungi. While we cannot cover all discoveries in this review article, we would like to highlight some recent discoveries that demonstrate this concept. In pathogenic fungi, effectors containing a conserved CFEM domain (common in several fungal extracellular membrane proteins) have been found to be over-represented in the genomes of pathogens [25]. These can contribute to virulence in many ways, but most recently asparagine-type CFEM effectors of *Verticillium* were found to support host colonization through the repression of host immune responses [26]. Within mutualistic fungi, the primary target of these effectors functionally characterized to date appear to be hormone-related proteins [9, 27, 28]. In all cases, hormone signaling was blocked by the presence of the effector protein. Production of toxic secondary metabolites is also a means by which many plants defend against invasion or foraging. While there are examples of effectors that target host enzymes in these pathways from parasitic nematodes [29] and bacteria [30], the recent literature has very little characterization of effectors from fungal pathogens that directly interact with, and modulate, host enzymatic function linked to secondary metabolism. In mutualistic fungi, only one effector protein has been identified to date that is able to alter host enzymatic activity through modulation of S-adenosylmethionine decarboxylase activity to promote polyamine accumulation [31]. Given the fact that metabolites like polyamines can inhibit the growth of other microbes, this mutualistic effector gives insight into one of the potential ways that mycorrhizal fungi can protect host roots from pathogens while simultaneously promoting their own colonization.

A fascinating development in recent years is the leveraging of comparative genomics to uncover the evolution (or conservation) of effector gene sequences to better understand host specialization of fungi, or investigate effector-like genes in lineages of fungi typically not dependent on a living host (e.g., saprotrophic fungi). Within smut fungi, this was used to investigate neofunctionalization of *Tin2* between *Ustilago maydis* and *S. scitamineum* [32]. This work demonstrated that, while the paralogues were able to interact with the same host kinase, the resulting impact on host range and metabolism was distinct. This would suggest that minor modifications in effector sequence led to different host specificity in these two pathogens in a manner reminiscent of oomycete pathogens [33]. In saprotrophic *Pseudozyma* yeasts, meanwhile, a significant number of effector-like sequences were found to be held in common with pathogenic fungi [34]. This included a functional *Pep1* sequence, a known virulence factor that inhibits the activity of plant peroxidases [35]. Taken together, these results would suggest that effector SSPs have under-explored roles in host specificity and that maintenance of these proteins from ancestral saprotrophic fungi suggests that effectors have unexpected roles in niche colonization (either a host niche or substrate colonization) for these fungi whereby certain stages of their lifecycle may require the ability to influence the physiology of other organisms.

## Effectors and the microbiome

**Fig. 2:**
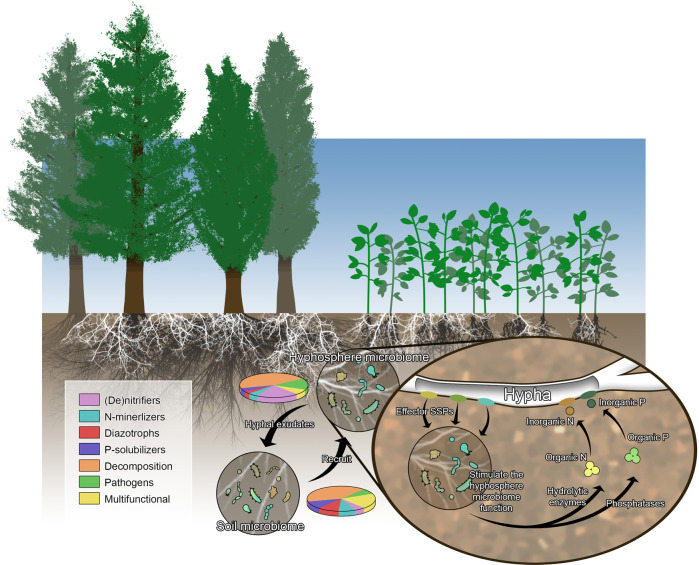
Small secreted proteins from soil-dwelling fungi may selectively influence the microbiome found within the hyphosphere. Theoretical framework representing how fungal secreted proteins and exudates may act on the microbiome to recruit specific microbes and alter microbiome functional composition (represented by the pie charts) or how SSPs secreted from fungal hyphae may stimulate the microbiome to assist in substrate breakdown and nutrient acquisition.

When a fungus colonizes a given niche, be that host tissues or a compartment within the growth substrate (e.g., soil), growth into the compartment must be coordinated across the whole fungal colony. Further, invading hyphae join a nascent microbiome and recent evidence has demonstrated that these hyphae are able to manipulate the community structure of these microbiomes in a non-random fashion that improves the function or fitness of the fungus (Fig. 2); [36–38]. The maintenance of SSPs bearing the hallmarks of effector proteins throughout the fungal tree of life, even in the most basal fungi studied to date [5], suggests that SSPs have roles that pre-date host colonization. Mounting evidence would suggest that one of the most basic functions of candidate effector SSPs in fungi is communication within a hyphal colony or in the manipulation of the surrounding microbiome.

Evidence for a role of effector proteins in regulating hyphal colony growth and aggregation comes from the genome annotation of the white rot fungus *Pleurotus ostreatus* [39]. In addition to secreted enzymes, this species also encodes a diverse array of small secreted proteins, one of which*, Small Secreted Protein 1*, was found to have a role in communication between hyphae and in coordinating the formation of fruiting bodies. Loss of the protein resulted in abnormal colony growth and delayed reproduction [39]. In the mutualistic fungus *L. bicolor*, the effector protein LbMiSSP8 was implicated in a role for both symbiosis and hyphal aggregation [40]. This protein, part of a larger family found in both mutualistic and saprotrophic fungi, is potentially one of the first functionally characterized examples of a protein family that maintains an ancestral role in hyphal dynamics and fruiting body formation that has also gained a novel function in symbiosis through neofunctionalization. As more genomes become available, similar situations appear common throughout entire fungal families [7, 8], divisions [41], and orders [5], whereby proteins initially characterized in pathogenic or mutualistic fungi as essential for host interactions also have homologs in saprotrophic fungi where they are hypothesized to have a more basal role pertaining to fungal physiology.

A newer understanding within fungi is also the concept that these candidate effector SSPs may be used to alter the microbiome of the hyphosphere or of plant tissues. This concept has been best advanced in the hemibiotrophic pathogenic model system of *Verticillium dahliae* where a series of effectors have been characterized as having dual roles in host manipulation and microbiome modulation [42, 43]. The first two effector proteins to be described from this system, VdAMP1 and VdAMP2, were found to have antibacterial activity, with the over-expression of VdAMP2 leading to increased spread of the fungus within a soil matrix [42]. More recently, VdAMP3 was found to repress bacteria that are normally antagonistic to *V. dahliae* within a host plant [43]. This latter protein was found to be part of a much larger protein family with homologs in the Ascomycetes, Basidiomycetes and early diverging fungi from the Mucoromycotina and Zoopagomycota. These results suggest that a role for SSPs in manipulating other microbes may be more prevalent than initially expected. While a similar mechanism in mutualistic fungi has not been demonstrated to date, it is likely that effectors from these fungi also exhibit a broader function in modifying microbial diversity and action within the hyphosphere. This is based on the observation that both ECM fungi [38] and AM fungi [36, 37] selectively alter their microbiome to supplement fungal nutrition (Fig. 2). While AM fungi use metabolites to achieve this [44], it is likely that effector proteins will also play a role.

## Effectors and the availability and acquisition of nutrients

Fungal nutrition is at the heart of the interaction between fungi and plant hosts or their environment, therefore nutrient availability is an important consideration in these interactions. In mutualistic symbioses, high levels of plant-available nutrients in the soil negatively affect root colonization, potentially linked to an increase in defensive signaling on the part of the plant host [45–47]. Within pathogenic interactions, nutrient-replete tissues are more vulnerable to biotrophic colonization, while deficient tissues are more vulnerable to necrotrophic pathogens, most notably observed with nitrogen level [48]. Thus, nutrient availability, both within the soil and plant tissues impacts the outcome of symbiosis, but how does it impact the secretome?

The nutritional status of the fungus, or its perception of nutrient availability in the external environment, is intimately linked to the expression of fungal secreted proteins [49]. Perhaps unsurprisingly, the majority of secreted proteins differentially regulated in response to limited nutrient availability are those involved in enzymatic degradation, such as proteases or cell-wall degrading enzymes [49]. As many biotrophic fungi also rely on host interactions to gain nutrients, however, it may be expected that nutrient limitation would also induce the expression of effectors to promote colonization of host tissues. While examples of this are rarer compared to regulation of other aspects of the secretome, there is evidence that fungal nutrient-sensing pathways and signaling do directly affect the expression of some effector genes [50–52]. For example, the expression of the *Cladosporium fluvum* effector Avr9 is induced by nitrogen deprivation and controlled by the nitrogen response regulator Nrf1, although the regulation of other effectors encoded by this pathogen remain nitrogen independent [52]. However, while expression of several nitrogen-regulated transcription factors has been linked to pathogenicity in several fungal plant pathogens, nutrient availability is unlikely to be one of the primary factors driving effector expression [53]. Comparisons of differential gene regulation in pathogens grown on nutrient-deficient media or *in planta* demonstrate that, while some candidate effectors are induced at low nutrient availability, the majority are only expressed in response to the plant host [54, 55].

As effector expression favors interactions with a host plant and results in the supply of nutrition to the fungus, it could be argued that most effectors indirectly affect fungal nutrient acquisition. However, some effectors more directly target host systems to improve nutrient flow. For example, some bacterial pathogens use transcriptional activator-like (TAL) effectors to induce the expression of plant sugar transporters (SWEET transporters; [56, 57]). In grapevine (*Vinus vinifera*), VvSWEET4 is similarly induced by the necrotrophic pathogen *Botrytis cinerea*, potentially through the action of fungal virulence factors [58], while in maize (*Zea mays*), the presence of the biotrophic pathogen *U. maydis* results in the upregulating of several plant and fungal sugar transporters to set up a sugar gradient to fungal sink tissues [59]. Overall, while these and other examples illustrate the connection between plant sugar transporter regulation and the presence of a colonizing fungus, the mechanisms behind these transcriptional changes are not well explored [60]. Likely, given the large number of effectors that still remain uncharacterized, there is high potential for future discovery of fungal effectors specifically targeting plant metabolism to encourage nutrient production.

## Conclusion

The exponential increase in available genomic resources in fungi is leading the study of effector and candidate effector proteins into new and very exciting directions. Overall, their role is now understood to be far more complex than simply direct interaction with a host immune system, but instead these proteins may also have functions in defining host range of symbiotic fungi, tailoring nutrient flows from host organisms, saprotrophic establishment, and guiding microbe-microbe interactions during niche colonization. Such a diversity in roles, however, will complicate efforts to functionally characterize these proteins. Comparative genomics should enable hypothetical functions to focus the efforts in this active area of research. Almost universally, the complement of effector SSPs appears to be increased in host-associated fungi [4, 25], suggesting that protein sequences with homologs in more basal fungi are likely to have roles targeting fungal physiology and microbiome modification. Meanwhile, duplicated effector sequences would be more likely to have undergone neofunctionalization to gain pivotal roles in the adaptation to new niches or host specificity. Therefore, using genome-guided studies, we can expect our understanding of this once enigmatic class of proteins to also grow exponentially in the coming years. The application of this knowledge will enable us to better leverage microbial function in a range of different managed and natural ecosystems.
